# Long non‐coding RNA LINC01225 promotes proliferation, invasion and migration of gastric cancer via Wnt/β‐catenin signalling pathway

**DOI:** 10.1111/jcmm.14627

**Published:** 2019-08-28

**Authors:** Ying Xu, Guohua Zhang, Chen Zou, Weidong Qi, Zhigang Gong, Guoliang Zhang, Gui Ma, Wenbo Zhang, Pengcheng Jiang

**Affiliations:** ^1^ Department of Laboratory Center Affiliated People's Hospital of Jiangsu University Zhenjiang China; ^2^ Department of General Surgery Affiliated People's Hospital of Jiangsu University Zhenjiang China

**Keywords:** epithelial‐mesenchymal transition (EMT), gastric cancer (GC), LINC01225, Wnt, β‐catenin

## Abstract

Emerging evidence has classified the aberrant expression of long non‐coding RNAs (lncRNAs) as a basic signature of various malignancies including gastric cancer (GC). LINC01225 has been shown to act as a hepatocellular carcinoma‐related gene, with its expression pattern and biological function not clarified in GC. Here, we verified that LINC01225 was up‐regulated in tumour tissues and plasma of GC. Analysis with clinicopathological information suggested that up‐regulation of LINC01225 was associated with advanced disease and poorer overall survival. Receiver operating characteristic (ROC) analysis showed that plasma LINC01225 had a moderate accuracy for diagnosis of GC. In addition, knockdown of LINC01225 led to retardation of cell proliferation, invasion and migration, and overexpression of LINC01225 showed the opposite effects. Mechanistic investigations showed that LINC01225 silencing inhibited epithelial‐mesenchymal transition (EMT) process and attenuated Wnt/β‐catenin signalling of GC. Furthermore, ectopic expression of Wnt1 or suppression of GSK‐3β abolished the si‐LINC01225‐mediated suppression against EMT, thereby promoting cell proliferation, invasion and migration of GC. In conclusion, LINC01225 promotes the progression of GC through Wnt/β‐catenin signalling pathway, and it may serve as a potential target or strategy for diagnosis or treatment of GC.

## 1 INTRODUCTION

Gastric cancer (GC) is the fifth most frequently diagnosed cancer and the third leading cause of cancer death worldwide.^1^ Although improvements in diagnostic tools and surgical techniques have been made in recent decades, the 5‐year overall survival for patients with GC remains unsatisfactory because of most patients with advanced or metastatic disease.^2^ Therefore, it is vital to discover effective biomarkers and targets for GC diagnosis and treatment.

Recent evidence from human genome sequencing has revealed that <3% of the human genome constitutes protein‐coding genes, while the majority of the genome transcribes vast numbers of non‐coding RNAs (ncRNAs).^3^ Long non‐coding RNAs (lncRNAs) are a class of ncRNAs that transcribe longer than 200 nucleotides, with limited or no protein‐coding capacity.^4^ Once considered as transcriptional noise, lncRNAs have recently been implicated in a wide range of biological processes including normal tissue development, regulation of cellular pluripotency, modulation of cell proliferation and apoptosis. Specifically, lncRNAs are emerging as key players in genetics and pathogenesis of cancer and their dysfunction is closely associated with cancer development, progression and metastasis including GC.^5^, ^6^ By acting as oncogenes or tumour suppressors, lncRNAs may contribute to GC tumorigenesis and progression. For example, metastasis‐associated lung adenocarcinoma transcript 1 (MALAT1) functions as a competing endogenous RNA (ceRNA) to attenuate the inhibitory effect of miR‐23b‐3p on autophagy‐related protein 12 (ATG12) and then leads to chemo‐induced autophagy and chemoresistance in GC.^7^ Moreover, long intergenic non‐coding RNA (lincRNA) FEZF1 antisense 1 (FEZF1‐AS1) represses p21 expression to promote GC proliferation through lysine‐specific demethylase 1(LSD1)‐mediated H3K4me2 demethylation.^8^ In contrast, lncRNA GAS5 enhances G1 cell cycle arrest via binding to YBX1 to regulate p21 expression in GC.^9^Our previous study showed that lncRNA MT1JP suppressed GC cell proliferation and migration by modulating miR‐214‐3p/RUNX3 axis.^10^ Given that the current understanding on GC‐associated lncRNAs may represent only the tip of iceberg, additional wok is still needed to further clarify the functional roles of many other lncRNAs in GC development.

Located at chromosome 1p35.2, LINC01225 is a lincRNA with a length of 2113 base pairs (bps).^11^ Wang et al first reported that LINC01225 acted as a hepatocellular carcinoma (HCC)‐related lncRNA and that its high expression was significantly associated with advanced TNM stage, larger tumour size and positive lymph node metastasis.^11^Specifically, they found that LINC01225 promoted HCC growth and invasion via epidermal growth factor receptor/mitogen‐activated protein kinase (EGFR/MAPK) pathway.^11^ However, the role of LINC01225 in other cancers including GC is unknown up to now and remains to be elucidated.

In this study, we firstly examined the expression pattern of LINC01225 in tumour tissues and plasma of GC. Our results demonstrated upregulation of LINC01225 in GC to be associated with advanced disease and poorer overall survival. Furthermore, LINC01225 promoted cell proliferation, motility and EMT potential of GC through the Wnt/β‐catenin signalling pathway. Collectively, our findings support an oncogenic role of LINC01225 in GC progression and suggest that LINC01225 may be served as a candidate biomarker for diagnosis, staging and outcome prediction of GC.

## 2 MATERIALS AND METHODS

### 2.1 Data extraction and analysis

The Cancer Genome Atlas (TCGA) and Gene Expression Omnibus (GEO) database were selected to learn the expression pattern of LINC01225 in GC initially. Raw RNA sequencing data containing 344 gastric adenoma and adenocarcinoma samples and 30 matched non‐cancerous samples were obtained from TCGA database. The data were then normalized and analysed with the edgeR package. Microarray dataset (GSE54129) containing transcriptome data of 111 GC samples and 21 non‐cancerous samples was downloaded from GEO database and normalized using Robust Multichip Average (RMA) method. The z‐score of log_2_ format of normalized data was used for further analysis.

### 2.2 Patients and specimens

A total of 109 pairs of GC tissues and adjacent non‐cancerous tissues were obtained from patients who had undergone radical gastrectomy in the Affiliated People's Hospital of Jiangsu University between 2015 and 2016 for this study. The adjacent normal tissues were obtained from tissues that were located 5 cm away from the edge of GC. All selected GC patients met the following inclusion criteria: (a) patients were newly diagnosed to have GC with definite pathological evidence; (b) no chemoradiotherapies were given before surgery. Plasma samples were collected from each subject, as well as 45 healthy donors included in the same period, and stored at −80°C before use. Tumour stage was evaluated according to the eighth TNM staging of the International Union against Cancer (UICC)/American Joint Committee on Cancer (AJCC) system.^12^ This study was approved by the Institutional Ethical Committee of Jiangsu University, and signed informed consents were obtained from all patients.

### 2.3 Cell culture and transfection

Five human GC cell lines SGC‐7901, MGC‐803, HGC‐27, AGS and BGC‐823 were obtained from the Institute of Biochemistry and Cell Biology of the Chinese Academy of Sciences (Shanghai, China). The human normal gastric epithelial cell line (GES‐1) was purchased from Procell Life Science & Technology. MGC‐803 was cultured with Dulbecco's modified Eagle's medium (DMEM; Thermo Fisher Scientific), while SGC‐7901, HGC‐27, AGS, BGC‐823 and GES‐1 cells were cultured with Roswell Park Memorial Institute (RPMI) 1640 medium (Thermo Fisher Scientific). All media were supplemented with 10% foetal bovine serum (FBS; Gibco), 100 U/mL penicillin and 100 μg/mL streptomycin (Gibco) in humidified air at 37°C with 5% CO_2_.

### 2.4 RNA isolation and reverse transcription‐quantitative polymerase chain reaction (RT‐qPCR)

Total RNA was extracted from tissues, plasma and different GC cells using TRIzol Reagent (Sangon Biotech). For RT‐qPCR, RNA was reversely transcribed into complementary DNA (cDNA) by using a Hieff First Strand cDNA Synthesis Super Mix for RT‐qPCR kit (Yeasen Biotech). Quantitative PCR analysis was carried out using the Hieff qPCR SYBR Green Master Mix kit (Yeasen Biotech) on ABI 7500 real‐time PCR system (AppliedBiosystems). The primer sequences were as follows: LINC01225 forward: 5′‐GTCCCTTACCTTGAGGTGCC‐3′, reverse: 5′‐CACGCCTTTGTGTTCTGGTG‐3′, Wnt1 forward: 5′‐ATCTTCGCTATCACCTCCGC‐3′, reverse: 5′‐GGCCGAAGTCAATGTTGTCG‐3′, GSK‐3β forward: 5′‐TGGTGCTGGACTATGTTCCG‐3′, reverse: 5′‐CCAACAAGAGGTTCTGCGGT‐3′, β‐catenin forward: 5′‐ACGAGCTGCTATGTTCCCTG‐3′, reverse: 5′‐ATTGCACGTGTGGCAAGTTC‐3′, Snail forward: 5′‐GCTCGAAAGGCCTTCAACTG‐3′, reverse: 5′‐GACATGGCCTTGTAGCAGCC‐3′, Slug forward: 5′‐TGTGACAAGGAATATGTGAGCC‐3′, reverse: 5′‐TGAGCCCTCAGATTTGACCTG‐3′, E‐cadherin forward: 5′‐GGTCTGTCATGGAAGGTGCTC‐3′, reverse: 5′‐CAGGATCTTGGCTGAGGATGG‐3′, N‐cadherin forward: 5′‐TCAACTGCAACCGTGTCTGT‐3′, reverse: 5′‐ATCGATCTGGGTCCTGAGCA‐3′, vimentin forward: 5′‐TGGACCAGCTAACCAACGAC‐3′, reverse: 5′‐GCCAGAGACGCATTGTCAAC‐3′, MMP7 forward: 5′‐GGCTTTAAACATGTGGGGCAA‐3′, reverse: 5′‐GGCCCATCAAATGGGTAGGA‐3′, myc forward: 5′‐GGACTTGTTGCGGAAACGAC‐3′, reverse: 5′‐CTCAGCCAAGGTTGTGAGGT‐3′, cyclin D1 forward: 5′‐AGGTCTGCGAGGAACAGAAG‐3′, reverse: 5′‐CCACGAACATGCAAGTGGC‐3′, β‐actin forward: 5′‐CATTCCAAATATGAGATGCGTTGT‐3′, reverse: 5′‐TGTGGACTTGGGAGAGGACT‐3′, U6 forward: 5′‐CTCGCTTCGGCAGCACA‐3′, reverse: 5′‐AACGCTTCACGAATTTGCGT‐3′. β‐actin or U6 was used as an endogenous control depending on the gene/sample detected. Relative expression levels were calculated by using the 2^−ΔΔCt^ method.

### 2.5 Synthesized oligos, constructs and treatment

Small interfering RNAs (siRNAs) were synthesized by GenePharm Company. The sequences of siRNAs targeting LINC01225 (si‐LINC01225) and GSK‐3β (si‐GSK‐3β) were presented as following: si‐LINC01225‐1 forward: 5′‐CCAGUUUCACUCCUGGUUUTT‐3′, reverse: 5′‐AAACCAGGAGUGAAACUGGTT‐3′, si‐LINC01225‐2 forward: 5′‐CCAUCAGGCCUUCUGCCAATT‐3′, reverse: 5′‐UUGGCAGAAGGCCUGAUGGTT‐3′, si‐GSK‐3β forward: GGACUAUGUUCCGGAAACATT, reverse: 5′‐UGUUUCCGGAACAUAGUCCTT‐3′. The LINC01225 and Wnt1 overexpression plasmids were also purchased from GenePharm. For transfection, the cells were grown in a 12‐well plate until confluence at 60%‐80% and were transfected with the indicated molecules with Lipofectamine 2000 (Thermo Fisher Scientific). For pharmacological inhibition of GSK‐3β activity, the cells were treated with 20 mmol/L lithium chloride (LiCl; Sigma‐Aldrich) as previously described.^13^ Equivalent dose of NaCl served as vehicle.

### 2.6 Cell counting, colony formation and apoptosis

For proliferation assay, GC cells were seeded into 24‐well plates at a density of 1 × 10^4^ cells/well. The cells were collected and counted everyday for 6 days. The results were plotted as cell growth curves. For colony formation assay, the cells were placed in 6‐well plates (1000 cells/well) and were cultured for 10 days. The colonies were then washed, fixed and stained with 0.5% crystal violet. Visible colonies were counted and photographed. For cell apoptosis assay, the indicated cells were stained with Annexin V‐FITC/PI cell apoptosis detection kit (BD Pharmingen) and analysed with a flow cytometer (BD FACSCalibur). Experiments were carried out in triplicate independently.

### 2.7 Migration and invasion assay

Migration and invasion assay was performed in transwell chambers without or with Matrigel‐coated membranes, respectively. Briefly, GC cells were seeded with serum‐free medium into the upper chambers at 5 × 10^4^ cells/well, and the bottom chambers contained medium with 10% FBS. After culture for 24 hours, cells on the upper surface were removed with a cotton swab and cells on the lower surface were stained and counted under a microscope.

### 2.8 Wound‐healing assays

Indicated cells were plated to confluence in 6‐well plates. Streaks across the plate were created in the monolayer with a pipette tip, followed by three washes in serum‐free medium. The cells were then cultured for 24 hours with medium containing 1% FBS. To ensure documentation of the same region, the wells were marked across the wounded area. Progression of migration was observed and photographed at 0 and 24 hours after wounding. The distance between the two edges of the scratch was measured and calculated.

### 2.9 Western blot

Equal amounts of protein samples were separated by 8%‐15% sodium dodecyl sulphate‐polyacrylamide gel electrophoresis (SDS‐PAGE) and transferred to nitrocellulose membranes. Bands were probed immunologically using anti‐Wnt1, GSK‐3β, GSK‐3β (Phospho‐Ser9), β‐catenin, β‐catenin (Phospho‐Ser37), β‐catenin (Phospho‐Thr41/Ser45), Snail, Slug, E‐cadherin, N‐cadherin, vimentin, MMP7, myc and cyclin D1 (EnoGene). GAPDH (Cell signalling) was probed as internal reference. Signals were detected using an enhanced chemiluminescence (ECL) system according to the manufacturer's instructions.

### 2.10 Immunohistochemistry

Briefly, sections were deparaffinized, rehydrated and incubated in sodium citrate buffer for antigen retrieval. Endogenous peroxidase was blocked by incubation in 3% H_2_O_2_ for 10 minutes at room temperature. The sections were then incubated with anti‐Ki‐67, E‐cadherin or N‐cadherin antibodies (EnoGene; 1:200 dilution) overnight at 4°C. Finally, HRP‐conjugated secondary antibody and diaminobenzidine (DAB) solution (Solarbio) was used to detect the signals. Slides were photographed under a microscope.

### 2.11 Tumour xenograft experiment

Four‐week‐old athymic BALB/c mice were purchased from Experimental Animal Center of the Chinese Academy of Sciences (Shanghai, China) and maintained under specific pathogen‐free conditions. The validated LINC01225 interference sequence was firstly subcloned into pLKO.1 vector to generate sh‐LINC01225 plasmid. SGC‐7901 cells stably transfected with sh‐LINC01225 or sh‐control were selected by puromycin (Sigma‐Aldrich). Approximately 2 × 10^6^ SGC‐7901 cells were re‐suspended with 0.2 mL phosphate buffer saline (PBS) and injected into the dorsal right flank of each nude mouse (six mice per group). Tumour volumes were calculated as 0.5 × length × width^2^ every 3 days. After 30 days, the mice were killed, and tumours were excised, weighed and immediately frozen at −80°C for future use. The procedures for animal studies were approved by the Animal Use and Care Committee of Jiangsu University.

### 2.12 Statistical analysis

All statistical analyses were conducted with SPSS 24.0 software (IBM, SPSS). Student's *t* tests or one‐way analysis of variance (ANOVA) was performed to determine the significance of differences between different groups. The associations between LINC01225 and the clinicopathological features were analysed by the Pearson chi‐squared test. Survival curves according to LINC01225 expression were generated using Kaplan‐Meier method, and their difference was evaluated by log‐rank test. Receiver operating characteristic (ROC) curve was established to evaluate the diagnostic value of LINC01225 for GC. A two‐sided *P* value < .05 was considered statistically significant.

## 3 RESULTS

### 3.1 Up‐regulation of LINC01225 correlated with advanced disease and worse outcome of GC

**Figure 1:**
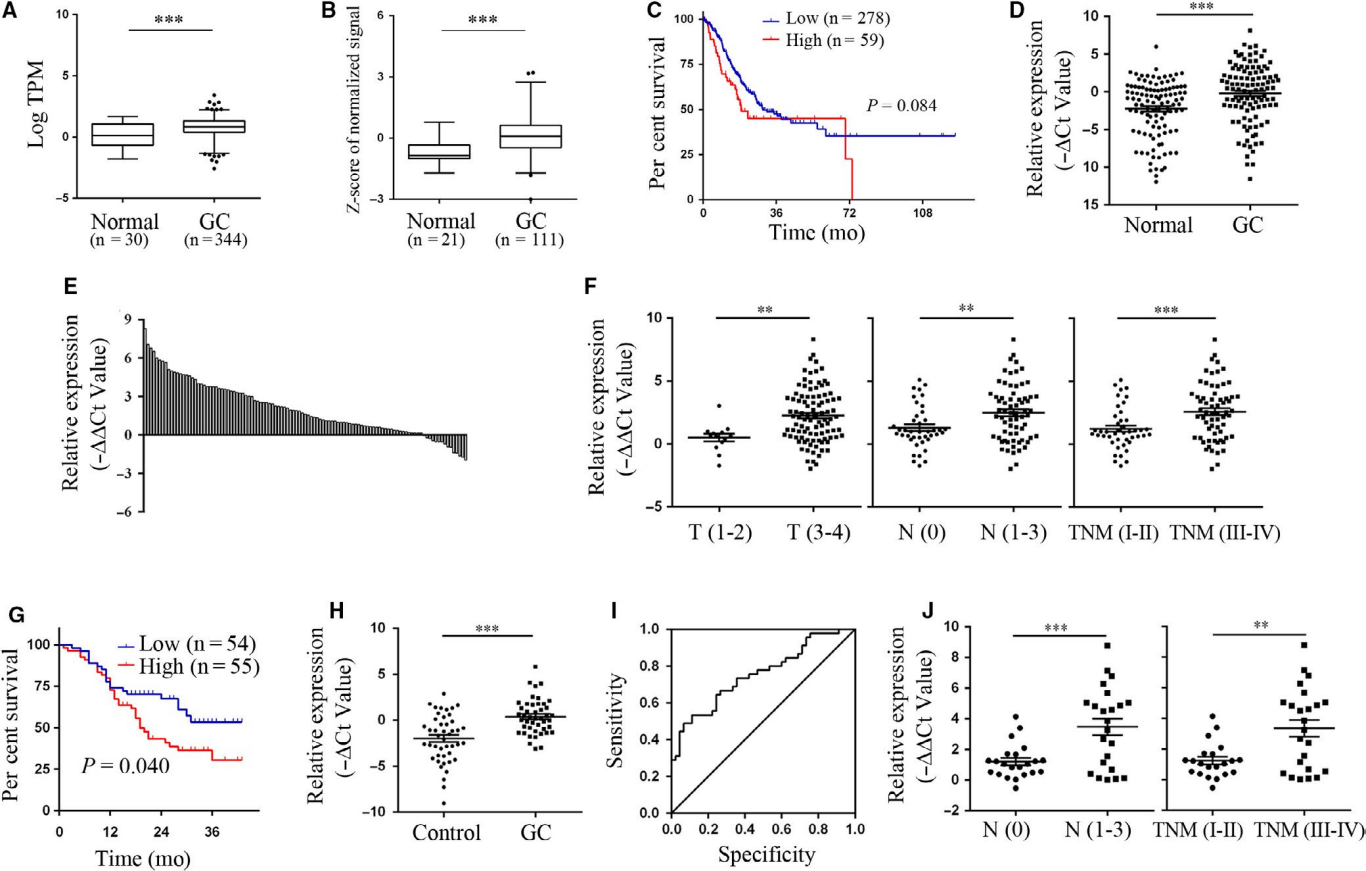
Up‐regulation of LINC01225 in tumour tissues and plasma of patients with GC. A, B, Analysis of LINC01225 expression levels in TCGA dataset and GEO dataset GSE54129. C, Kaplan‐Meier analysis for overall survival in TCGA dataset according to the mean expression level of LINC01225. D, E, RT‐qPCR for the expression of LINC01225 in GC tissues and adjacent normal tissues. F, The correlation between tissue LINC01225 expression and invasion depth, lymphatic metastasis or TNM stage. G, Kaplan‐Meier analysis for overall survival according to the median expression level of LINC01225. H, The expression levels of plasma LINC01225 in patients with GC and healthy controls. I, The ROC curve for plasma LINC01225 on diagnosis of GC. J, The correlation between plasma LINC01225 expression and lymphatic metastasis or TNM stage. **P < .01, ***P < .001

To understand the expression pattern of LINC01225 in GC, we compared the data of transcripts per million reads (TPM) between 344 GC samples and 30 matched non‐cancerous samples from TCGA database. The results showed that LINC01225 expression level of GC samples was increased by 5.23‐fold compared to that of normal samples (Figure 1A). Consistently, LINC01225 expression level was up‐regulated in GSE54129 dataset (Figure 1B). In addition, Kaplan‐Meier analysis showed that patients with higher expression of LINC01225 tended to have shorter overall survival time than those with lower expression of LINC01225 in TCGA database (Figure 1C). To validate these findings, we then detected LINC01225 expression levels in a cohort of 109 paired GC tissues and adjacent non‐cancerous tissues by RT‐qPCR. As shown in Figure 1D, the expression levels of LINC01225 were significantly higher in tumour tissues than those in adjacent non‐cancerous tissues. We further found that 65 cases displayed at least twofold increase in GC tissues compared with the paired non‐cancerous tissues (Figure 1E). Importantly, up‐regulation of LINC01225 was significantly correlated with advanced TNM stage, increased invasion depth and positive lymphatic metastasis (Table 1 & Figure 1F). In addition, smoke was significantly associated with increased LINC01225 level (Table 1).Consistent with the TCGA findings, we also observed a worse overall survival for patients with high LINC01225 expression (Figure 1G).

**Table 1:** Correlation between LINC01225 expression and patients' clinicopathological characteristics

Features	Number	LINC01225 expression		*P* value
		High	Low	
Gender				
Male	81	44	37	.170
Female	28	11	17	
Age (y)				
≤65	53	25	28	.504
>65	56	30	26	
Location				
Proximal	54	31	23	.224
Middle	27	10	17	
Distal	28	14	14	
Differentiation				
Moderately	47	27	20	.204
Poorly	62	28	34	
Size (cm)				
≤5 cm	73	36	37	.734
>5 cm	36	19	17	
TNM stage				
I‐II	44	13	31	<**.001**
III‐IV	65	42	23	
Invasion depth				
T1‐2	13	1	12	**.001**
T3‐4	96	54	42	
Lymphatic metastasis				
Negative	41	13	28	**.002**
Positive	68	42	26	
Distant metastasis				
M0	107	53	54	.495
M1	2	2	0	
Smoke				
NO	76	32	44	**.008**
Yes	33	23	10	
Drink				
NO	83	40	43	.398
Yes	26	15	11	

The bold values mean *P* < .05, that indicates the difference reach statistical signifcance.

To further explore the potential utility of LINC01225 for GC diagnosis, we detected the expression of LINC01225 in plasma samples from 45 randomly selected GC patients and paired healthy donors. The stability of circulating lncRNA has been well documented in previous studies.^10^, ^11^ Herein, we found that the expression levels of plasma LINC01225 were significantly higher in GC patients than those in healthy controls (Figure 1H). Up‐regulation of plasma LINC01225 was significantly associated with advanced TNM stage and positive lymphatic metastasis, respectively (Figure 1J). Finally, plasma LINC01225 had an area under the ROC curve (AUC) of 0.755 (95% CI, 0.655‐0.854, *P* < .001) for diagnosis of GC (Figure 1I). These findings suggest that LINC01225 expression in tissue or plasma may be a candidate predictor for GC diagnosis and staging assessment.

### 3.2 LINC01225 knockdown inhibited proliferation, migration and invasion of GC cells

**Figure 2:**
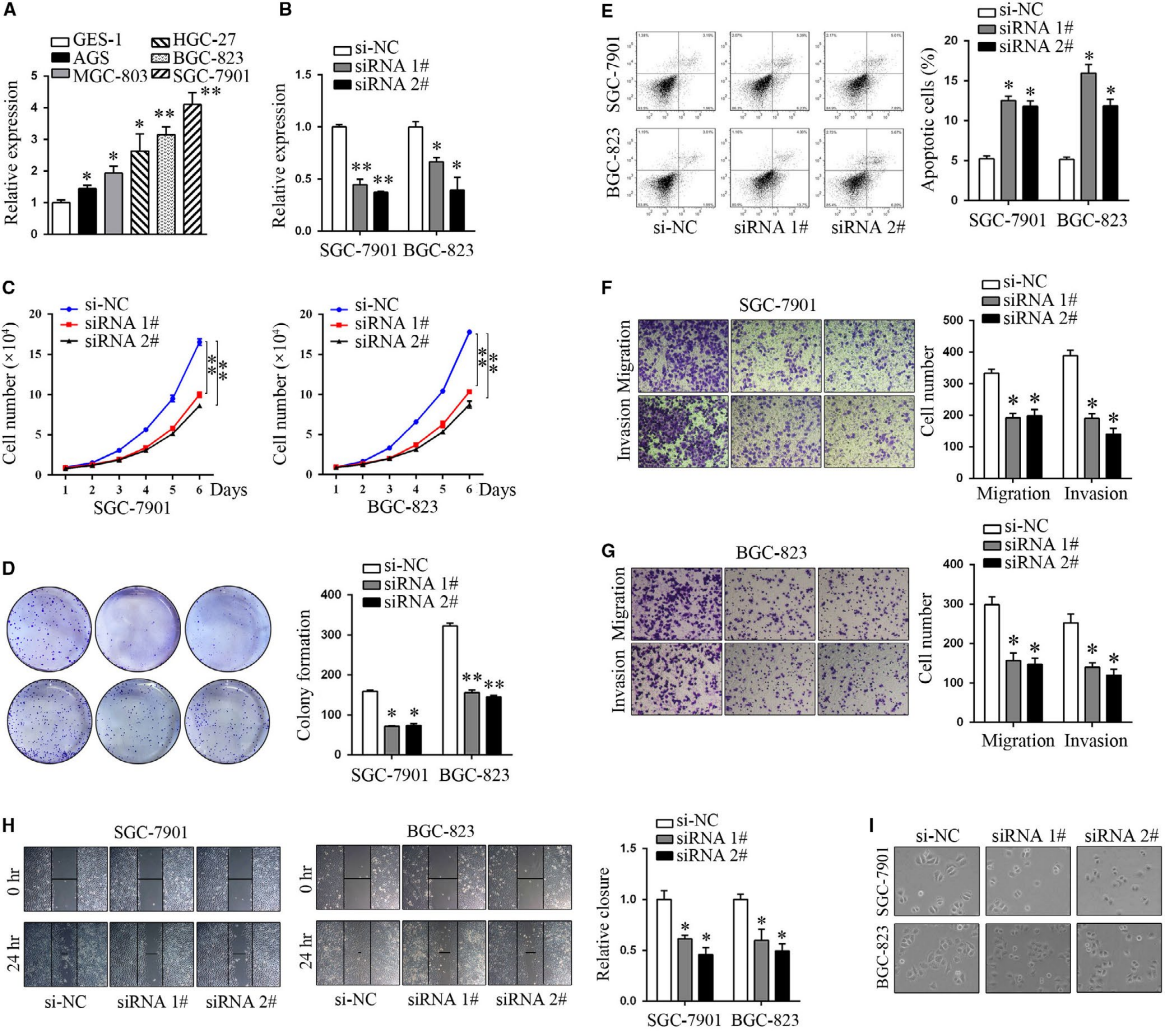
Knockdown of LINC01225 suppressed proliferation, migration and invasion of GC cells. A, The expression profile of LINC01225 in diverse GC cells compared to GES‐1 cells. B, Knockdown efficiency of the indicated siRNA oligos targeting LINC01225. C, D, Cell proliferation of GC cells transfected with si‐LINC01225 oligos was measured by cell counting assays and colony formation assays. D, Apoptosis of LINC01225‐silencing GC cells. F‐H, The motility ability of GC cells transfected with si‐LINC01225 was determined by wound‐healing assays and transwell assays. I, The morphology of GC cells transfected with si‐LINC01225 was visualized by phase‐contrast microscopy. *P < .05, **P < .01

To investigate the roles of LINC00978 in GC, we firstly profiled its expression in a panel of GC cell lines (MGC‐803, AGS, HGC‐27, BGC‐823 and SGC‐7901). Our data showed that LINC01225 was generally up‐regulated in the GC cell lines compared with that in GES‐1 (Figure 2A). Next, we designed two independent siRNAs targeting LINC01225 and validated their knockdown efficiency in SGC‐7901 and BGC‐823 cells (Figure 2B). Cell counting assays and colony formation assays consistently showed that LINC01225 knockdown led to growth retardation of SGC‐7901 and BGC‐823 cells (Figure 2C,D). In addition, LINC01225 silencing induced a significant increase in the percentage of apoptotic cells in both SGC‐7901 and BGC‐823 cells(Figure 2E). We then tested the motility of GC cells by wound‐healing assays and transwell assays. The results showed that LINC01225 silencing decreased the migration and invasion ability of GC cells (Figure 2F‐H). In contrast, overexpression of LINC01225 in AGS cells showed the opposite effects, as represented by increase in cell proliferation, migration and invasion, and by decrease in apoptosis (Figure S1A‐E). Moreover, knockdown of LINC01225 in SGC‐7901 and BGC‐823 cells rendered a mesenchymal‐epithelial transition (MET) in cell morphology (Figure 2I). Taken together, our findings suggest that LINC01225 may enhance GC progression by promoting proliferation, invasion and migration and by inhibiting of apoptosis.

### 3.3 LINC01225 knockdown suppressed epithelial‐mesenchymal transition (EMT) of GC

**Figure 3:**
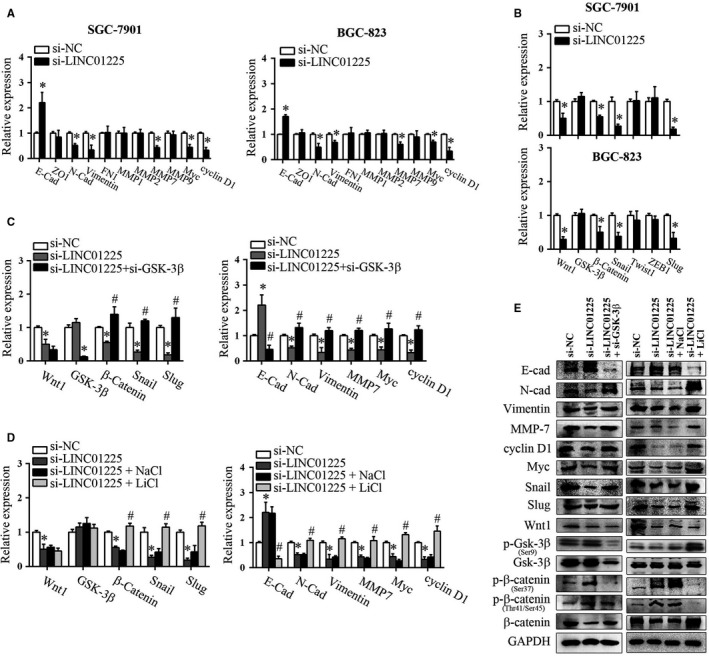
LINC01225 regulated EMT process through Wnt/β‐catenin signalling pathway. A, RT‐qPCR analysis of the main EMT markers E‐cad, ZO1, N‐cad, vimentin, FN1, MMP1, MMP2, MMP7, MMP9 as well as myc and cyclin D1 in LINC01225‐silencing GC cells. B, RT‐qPCR analysis of the main EMT‐inducing transcription factors Snail, Twist, ZEB1, Slug as well as Wnt‐related genes in LINC01225‐silencing GC cells. LINC01225‐silencing GC cells were co‐transfected with si‐GSK‐3β, or treated with GSK‐3β inhibitor LiCl. Relative expression of EMT markers and Wnt‐related genes were determined by RT‐qPCR (C, D) or Western blot (E). *P < .05 vs si‐NC, #*P* < .05 vs si‐LINC01225

Since EMT plays a crucial role in cancer progression by conferring an invasive phenotype,^14^ we then asked whether LINC01225 affected the EMT process of GC. To test this, the expression of main epithelial and mesenchymal markers in SGC‐7901 and BGC‐823 cells was examined upon LINC01225 knockdown. As shown in Figure 3A,E, LINC01225 silencing down‐regulated the expression of N‐cadherin, vimentin and MMP‐7, and up‐regulated the expression of E‐cadherin both at mRNA and protein levels. We next detected the expression of EMT‐inducing transcription factors and consistently found that LINC01225 silencing led to remarkable decrease in the mRNA and protein levels of Snail and Slug (Figure 3B,E). These findings suggest that LINC01225 crucially regulates the EMT process. Although ZO‐1, FN1, MMP1, MMP2, MMP7, Twist and ZEB1 are also critical markers or transcription factors involved in EMT, our results revealed that LINC01225 had minimal impact on these molecules.

### 3.4 Wnt/β‐catenin signalling contributed to LINC01225‐mediated EMT process

We then asked the mechanisms by which LINC01225 regulated EMT process of GC cells. Previous work has revealed that many growth factors, such as Wnt, transforming growth factor‐β(TGF‐β) and Notch, are engaged to trigger and complete an EMT process.^15^ Specifically, a close interplay between Wnt signalling and cadherin‐mediated cell adhesion has been supported by profound evidence.^16^ Previous studies have also provided evidence that many lncRNAs such as LINC01133, LINC01197 and SLCO4A1‐AS1 may affect EMT through direct or indirect regulation of Wnt signalling.^17^, ^18^, ^19^ Here, we examined Wnt‐related genes by LINC01225 knockdown and found that Wnt signalling was distinctly suppressed, as represented by decreased mRNA and protein expression of Wnt1 and β‐catenin(Figure 3B,E). These data suggest that Wnt signalling acts downstream of LINC01225.

Activation of Wnt signalling can induce GSK‐3β inhibition, reversal of β‐catenin phosphorylation and thereby promote β‐catenin accumulation and nuclear relocation.^20^ We observed that si‐LINC01225‐induced decrease in Wnt1 did not alter the expression or Ser9 phosphorylation of GSK‐3β (Figure 3E), indicating that LINC01225 silencing mainly affected the activity of GSK‐3β. This is in line with the reports that Wnt‐dependent GSK‐3β inhibition relies on the direct interaction of GSK‐3β with the scaffold protein Axin and the associated protein adenomatous polyposis coli (APC) rather than the expression or serine phosphorylation of GSK‐3β.^21^, ^22^ We further determined the activity of GSK‐3β by measuring the phosphorylation of β‐catenin and found that LINC01225 silencing restored β‐catenin phosphorylation at Ser37/Thr41/Ser45 and destabilized β‐catenin (Figure 3E). Decreased β‐catenin by LINC01225 knockdown further attenuated the expression of β‐catenin target genes including cyclin D1 and myc besides mesenchymal markers (Figure 3E). We then asked whether activation of Wnt signalling could reverse the si‐LINC01225‐mediated suppression against EMT. To test this, LINC01225‐silencing SGC‐7901 cells were co‐transfected with si‐GSK‐3β, or treated with LiCl, a widely used GSK‐3β inhibitor.^23^ As expected, we found that either genetic knockdown or pharmacological inhibition of GSK‐3β constrained the expression of epithelial marker E‐cadherin and enhanced the expression of mesenchymal markers including N‐cadherin, vimentin and MMP‐7 (Figure 3C‐E). Consistently, the expression levels of β‐catenin, Snail and Slug were all recovered by GSK‐3β inhibition. Furthermore, we tested whether overexpression of Wnt1 had similar effects as inhibition of GSK‐3β exerted. As expected, ectopic expression of Wnt1 also reversed si‐LINC01225‐mediated EMT suppression in SGC‐7901 cells (Figure S2A‐C).Therefore, these findings support that Wnt/β‐catenin signalling contributes to LINC01225‐mediated EMT process.

### 3.5 Inhibition of GSK‐3β counteracted the tumour suppressive effect by LINC01225 silencing

**Figure 4:**
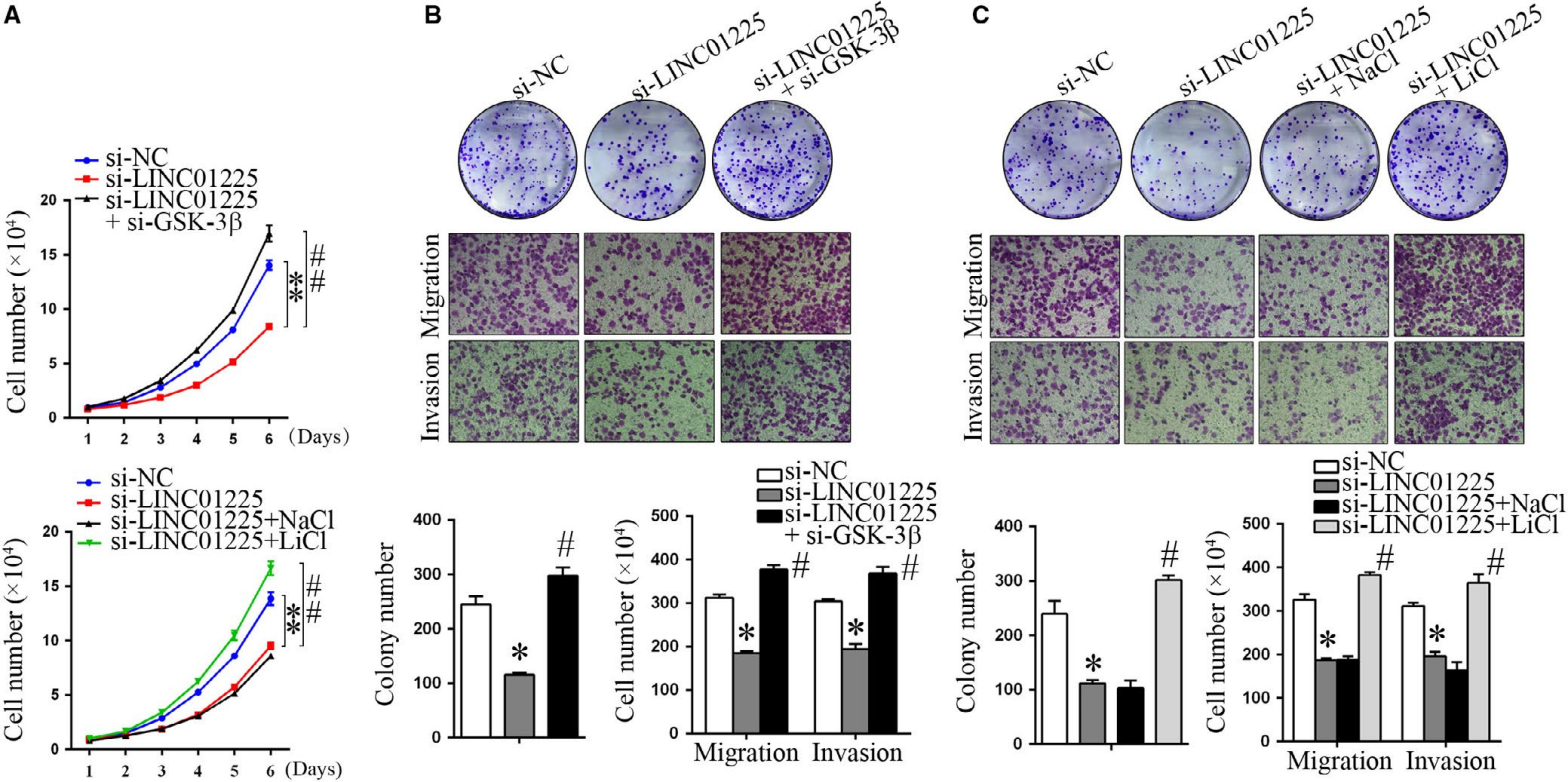
Inhibition of GSK‐3β counteracted the tumour suppressive effect by LINC01225 silencing. LINC01225‐silencing SGC‐7901 cells were co‐transfected with si‐GSK‐3β, or treated with LiCl. A, Cell counting assays. B, C, Colony formation assays, migration and invasion assays. *P < .05, **P < .01 vs si‐NC. #*P* < .05, ##*P* < .01 vs si‐LINC01225

Since Wnt signalling acted downstream of LINC01225 to promote EMT process, we then asked whether Wnt signalling was involved in LINC01225‐mediated phenotype change in GC. To test this, LINC01225‐silencing SGC7901 cells were co‐transfected with si‐GSK‐3β or treated with LiCl and cell proliferation and motility were investigated. As expected, si‐LINC01225‐mediated inhibition of cell proliferation, migration and invasion was completely reversed by si‐GSK‐3β or LiCl (Figure 4A‐C).

### 3.6 LINC01225 silencing suppressed GC tumorigenesis in vivo

**Figure 5:**
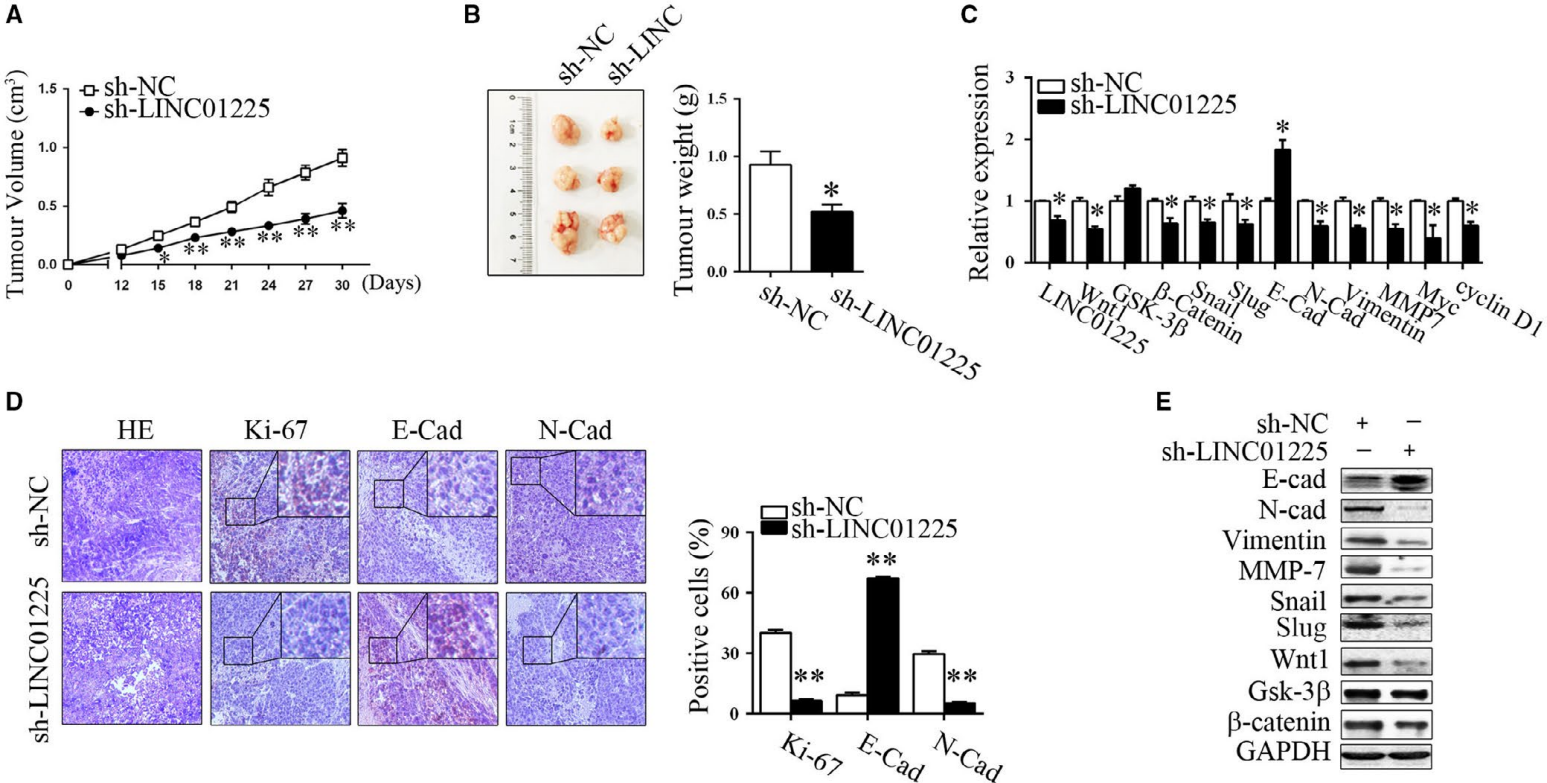
LINC01225 silencing suppressed GC tumorigenesis in vivo. A, B, Growth curve and weight of xenograft tumours from sh‐LINC01225 and sh‐NC groups. C, RT‐qPCR analysis for EMT and Wnt‐related genes in tumour tissues. D, Representative images of HE staining and immunohistochemical staining of Ki‐67, E‐cad and N‐cad. E, Representative blots of EMT and Wnt‐related genes. *P < .05, **P < .01

The effect of LINC01225 on GC tumorigenesis in vivo was determined by using a subcutaneous xenograft tumour model. As shown in Figure 5A,B, knockdown of LINC01225 significantly suppressed tumour growth and tumour weight. Accordingly, LINC01225 silencing decreased the expression of N‐cadherin, vimentin, MMP‐7, myc, cyclin D1, Wnt1, β‐catenin, Snail and Slug, and increased the expression of E‐cadherin, as evaluated by RT‐qPCR, immunohistochemistry or Western blot assays (Figure 5C‐E). Consistently, sections from sh‐LINC01225 group had less Ki‐67‐positive proliferating cells than those from sh‐NC group (Figure 5D). In summary, these data indicate that LINC01225 silencing can suppress GC growth, EMT process and Wnt signalling in vivo.

## 4 DISCUSSION

LncRNAs have emerged as critical players in tumorigenesis and cancer progression; however, the potential roles and mechanistic details for most lncRNAs still remain unclear in GC. Herein, we observed that LINC01225 was up‐regulated in both in tumour tissues and plasma from GC patients, and this upregulation was closely correlated with advanced TNM stage, increased invasion depth, positive lymphatic metastasis and poorer overall survival. In addition, plasma LINC01225 expression enabled the discrimination of GC patients from healthy controls with an AUC of 0.755. Therefore, LINC01225 may serve as a potential indicator for GC diagnosis, staging and outcome prediction.

Regarding the biological functions of LINC01225 in GC, we employed gain and loss of function techniques and studied the cell proliferation, apoptosis and motility of GC cells. The results showed that knockdown of LINC01225 in GC cells resulted in retardation of proliferation, migration and invasion and induction of apoptosis in vitro, while overexpression of LINC01225 led to the opposite effects. These findings basically suggested an oncogenic role of LINC01225 and were further supported by in vivo studies in which LINC01225 knockdown decreased tumour growth and reduced tumour weight.

Local progression and metastasis are responsible for as much as 90% of cancer‐induced mortality,^24^ and EMT is considered to be critical for tumour progression in GC. The process of EMT is characterized by changes of cell polarity and cell‐cell adhesion for epithelial cells and is associated with the downregulation of epithelial markers and aberrant upregulation of mesenchymal markers.^25^ Many lncRNAs have recently been demonstrated to play an essential role in regulating EMT.^26^ For example, lnc‐ATB (lncRNA activated by TGF‐β) can mediate TGF‐β‐induced EMT and is shown to promote metastasis in various cancers.^27^ Similarly, Chen et al indicated that MANCR (MALAT2‐activated lncRNA) contributes to GC migration by inducing EMT via the MEK/ERK pathway.^28^ In this study, we found that LINC01225 knockdown reduced N‐cadherin, vimentin and MMP‐7 expression and increased E‐cadherin expression, suggesting that LINC01225 crucially regulated the EMT process of GC cells. Consistently, EMT‐inducing transcription factors including Snail and Slug were also down‐regulated by LINC01225 silencing.

To further address the mechanisms by which LINC01225 regulated EMT process of GC cells, we focused on Wnt/β‐catenin signalling pathway. The importance of Wnt signalling on malignant cancer progression and EMT was supported by several lines of evidence. Firstly, many target genes of Wnt signalling such as Twist and Slug directly inhibit the E‐cadherin gene promoter.^29^, ^30^ Wnt target genes also encode ZEB1, vimentin, MMPs or the cell adhesion molecule L1.^31^, ^32^ In addition, loss of cadherin‐mediated cell adhesion can in turn promote β‐catenin release and accumulation,^33^ suggesting a close interplay between cadherin‐mediated cell adhesion and Wnt signalling. In the absence of Wnt, the cytoplasmic β‐catenin protein is constantly phosphorylated by the action of AXIN complex.^21^ This phosphorylation event targets β‐catenin for proteasome‐mediated proteolytic degradation. When Wnt proteins bind the cell‐surface receptors, GSK3‐dependent β‐catenin phosphorylation is suppressed and β‐catenin is stabilized. Here, we observed that LINC01225 silencing led to decrease in Wnt1 expression and thereby restored β‐catenin phosphorylation at Ser37/Thr41/Ser45 and destabilized β‐catenin in SGC‐7901 cells. As a result, β‐catenin target genes including cyclin D1, myc, Slug, MMP7 and vimentin were consistently restrained by LINC01225 silencing. These findings were generally replicable in the subsequent xenograft tumour study. Further, activation of Wnt signalling either by overexpression of Wnt1 or by suppression of GSK‐3β reversed the si‐LINC01225‐mediated inhibition against EMT. Thus, it is unsurprised that inhibition of GSK‐3β promoted proliferation, invasion and migration of GC cells.

Although our data showed that LINC01225 was critically involved in Wnt/β‐catenin signalling and thereby promoted EMT and malignant progression of GC, it is still unclear about how LINC01225 regulates these genes. Despite that lncRNAs are characterized by the complexity of their mechanisms in relation to Wnt signalling, previous studies have provided several constructive and insightful models to inspire further research. These models may be generally summarized by lncRNA's interaction with DNA, RNA and proteins. For example, MALAT1 can bind to CTNNB1 promoter region and recruit methyltransferase to promote CTNNB1 promoter methylation, thereby inhibiting CTNNB1 and modulating the Wnt signalling pathway.^34^ Besides, LINC01197 restrains Wnt/β‐catenin signalling by disrupting the interaction between β‐catenin and TCF4 in pancreatic ductal adenocarcinoma.^17^ In addition, LINC01133 directly targets miR‐106a‐3p and in turn inhibits GC metastasis through inactivating the APC/Wnt/β‐catenin pathway.^19^ Therefore, ongoing work is strongly needed to determine the localization of LINC01225 and to clarify the DNA binding potential, the ceRNA potential or the protein binding potential of LINC01225 with regard to Wnt signalling. This is also the focus of our future research.

In conclusion, our results showed that the expression of LINC01225 was increased in GC and its high expression correlated with the disease malignancy. LINC01225 promoted proliferation, migration, invasion and EMT process of GC cells through Wnt/β‐catenin signalling pathway. Our findings not only provide novel evidence for LINC01225‐mediated progression of GC but also suggest a potential target or strategy for diagnosis or treatment of GC.

## CONFLICT OF INTEREST

The authors declare that they have no conflicts of interest.

## AUTHORS CONTRIBUTIONS

WZ and PJ designed the study. YX, GZ, CZ, WQ and WZ performed experiments. YX, GZ, GZ, GM, ZG and WZ analysed the data. YX, GZ, GM, WZ, and PJ interpreted the results. YX, GZ, WZ and PJ wrote the manuscript. All authors have read and approved the final submitted manuscript.
