# Supplementary figures and images for: Long non‐coding RNA LINC01225 promotes proliferation, invasion and migration of gastric cancer via Wnt/β‐catenin signalling pathway
Source: J Cell Mol Med. 2019 Aug 28;23(11):7581–91. doi: 10.1111/jcmm.14627 (PMC6815774; doi:10.1111/jcmm.14627)

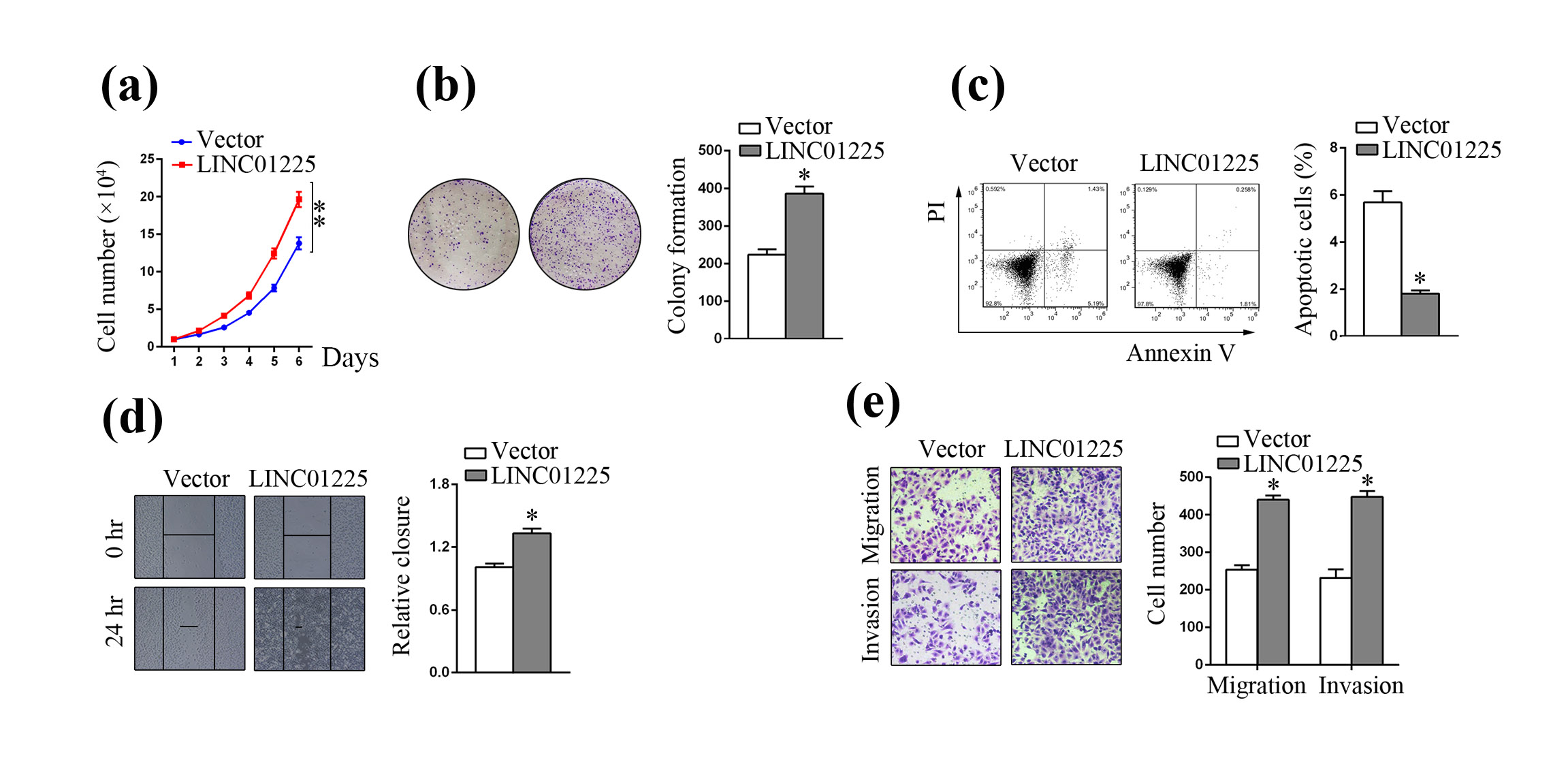

Supplement: Supplementary file 1 [file JCMM-23-7581-s001.tiff]

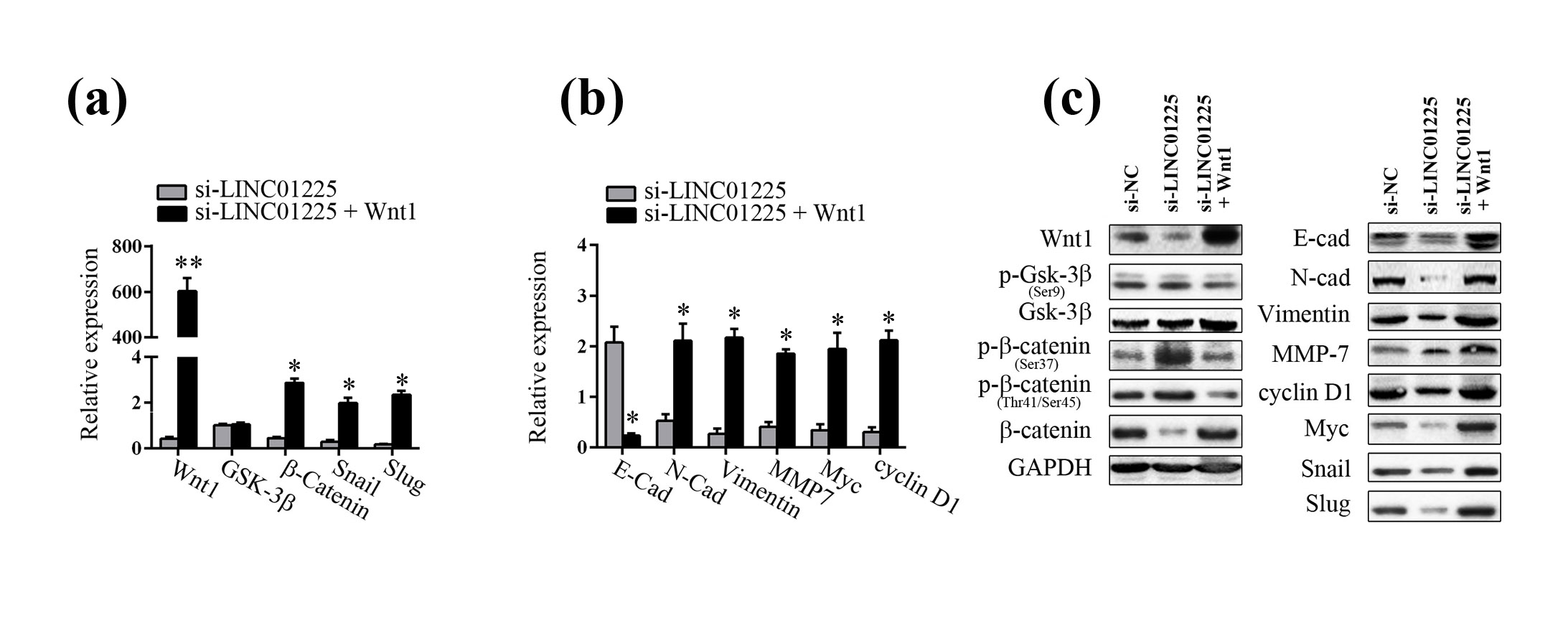

Supplement: Supplementary file 2 [file JCMM-23-7581-s002.tiff]
